# Paracingulate Sulcus Asymmetry in the Human Brain: Effects of Sex, Handedness, and Race

**DOI:** 10.1038/srep42033

**Published:** 2017-02-14

**Authors:** Xuehu Wei, Yan Yin, Menglin Rong, Jinfeng Zhang, Lijie Wang, Yan Wu, Qing Cai, Chunshui Yu, Jiaojian Wang, Tianzi Jiang

**Affiliations:** 1 Key Laboratory for NeuroInformation of the Ministry of Education, School of Life Science and Technology, University of Electronic Science and Technology of China, Chengdu, 625014, China; 2 Key Laboratory of Brain Functional Genomics, Ministry of Education, Shanghai Key Laboratory of Brain Functional Genomics, School of Psychology and Cognitive Science, East China Normal University, Shanghai, 200062, China; 3 Department of Radiology, Tianjin Medical University General Hospital, Tianjin 300052, China; 4 Brainnetome Center, Institute of Automation, Chinese Academy of Sciences, Beijing, 100190, China; 5 National Laboratory of Pattern Recognition, Institute of Automation, Chinese Academy of Sciences, Beijing, 100190, China; 6 CAS Center for Excellence in Brain Science and Intelligence Technology, Institute of Automation, Chinese Academy of Sciences, Beijing, 100190, China; 7 The Queensland Brain Institute, the University of Queensland, Brisbane, QLD 4072, Australia

## Abstract

The anterior cingulate cortex (ACC), which is thought to play a key role in cognitive and affective regulation, has been widely reported to have a high degree of morphological inter-individual variability and asymmetry. An obvious difference is in the morphology of the paracingulate sulcus (PCS). Three types of PCS have been identified: prominent, present, and absent. In this study, we examined the relationship between PCS asymmetry and whether the asymmetry of the PCS is affected by sex, handedness, or race. PCS measurements were obtained from four datasets. The statistical results revealed that the PCS was more often prominent and present in the left hemisphere than in the right. The percentage of right-handed males with a prominent PCS was greater than that of right-handed females, but the percentage of left-handed males with a prominent PCS was lower than that of left-handed females. In addition, both male and female and both left-handed and right-handed subjects showed a leftward asymmetry of the PCS. Furthermore there were no significant racial differences in the leftward asymmetry of the PCS. Our findings about the morphological characteristics of the PCS may facilitate future clinical and cognitive studies of this area.

The cingulate cortex, which is located bilaterally in the medial frontal lobes, is the most prominent structure of the human brain medial wall. In particular, its anterior portion has been widely investigated to decipher its complex functions. The anterior cingulate cortex (ACC), which includes the Brodmann areas (BA) 24, 25, and 33 and covers the corpus callosum^1^, plays a key part in many cognitive or executive functions such as emotion, motor behaviors, memory, and learning^2^. In addition, postmortem and functional imaging studies suggested that dysfunction of the ACC is related to the pathophysiology of schizophrenia^3^. The main sulcus in the ACC is the cingulate sulcus (CS), which runs along the corpus callosum and extends posteriorly into the parietal lobe as the marginal ramus. From the dorsal to the anterior portion of the CS, a sulcus that is often present and runs parallel to the CS was referred to by Elliot Smith as the paracingulate sulcus (PCS)^4^. A postmortem study^5^ showed that the presence of a PCS is associated with a relative expansion of the paralimbic ACC, which corresponds to BA 24c and BA 32. A previous study found a negative association between the PCS and the CS, when both the PCS and CS were present; specifically, a larger paracingulate was associated with a smaller cingulate volume^6^. The presence of a PCS was associated with an approximate mean increase of 88% in the paralimbic volume and an approximate mean decrease of 39% in the ACC volume^7^. Recently, the relationship between PCS variability and cortical morphometry in healthy humans has been investigated^8^,^9^ and showed that the presence of the PCS was related to the volumes of the ACC and the paracingulate cortex.

The existence of the PCS shows significant individual and hemispheric differences. The PCS is not always present in both cerebral hemispheres. Based on the criteria for the definition of the appearance of the PCS in a healthy population^10^,^11^, the PCS can be classified into three categories: prominent, present, and absent. The PCS can be described as “prominent” if it is longer than 40 mm, as “present” if it extends more than 20 mm, and as “absent” if the length is less than 20 mm. Following these criteria, Paus and colleagues^6^ examined 247 young healthy volunteers’ brain images obtained by magnetic resonance imaging (MRI) and found a higher incidence of the PCS in the left hemisphere than in the right as well as a significant effect of sex in the distribution of the prominent, present, and absent PCS in males and females. Female subjects tended to present either a well-developed PCS or lacked a PCS, whereas the intermediate type of PCS was quite common in males. Subsequently, Stuart and colleagues found the same result; specifically, a prominent PCS was more common in the left hemisphere than in the right^10^. Furthermore, leftward asymmetry of the PCS that is greater in adult males than females has been consistently demonstrated by classifying the PCS into predominant, present, and absent^6^,^10^,^12^,^13^. Thus, the PCS is present in many healthy populations and is more commonly found (and more prominent) in the left hemisphere. Furthermore, the appearance of the PCS has been shown to be related to the cytoarchitecture^5^, volume^6^,^7^, and functions^14^,^15^,^16^,^17^ of this region in both healthy populations and schizophrenia patients. Clark and colleagues found that the leftward asymmetry of the PCS correlated with semantic verbal fluency in a group of controls but not in a group of adolescent-onset psychosis patients^18^. Another study reported that leftward asymmetry of the PCS correlated positively with the spatial component of working memory^19^. Although several previous studies revealed asymmetry in the PCS between the left and right hemispheres, whether the PCS distribution patterns of the prominent, present, and absent PCS is affected by handness and race in males and females is still largely unknown.

The goal of the current study was to elucidate the relationship between the appearance of the PCS and the effects of sex, handedness, and race by examining the morphology of the PCS in each hemisphere using structural MRIs from each subject in four datasets. Three datasets contained right-handed subjects and the fourth dataset comprised left-handed subjects for sex, handness, and race related studies.

## Results

### Hemispheric asymmetry

The numbers and frequencies for all combinations of the left versus the right PCS morphology in the overall sample and each separate sample from datasets 1–4 are shown in Table 1. The chi-square statistical finding for each dataset identified a significant asymmetry of the PCS in the left and right hemispheres (dataset 1: 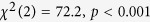, dataset 2: 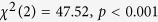, dataset 3: 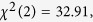

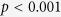
, dataset 4: 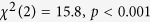). In addition, McNemar’s test of the overall samples for hemispheric asymmetry was also identified as significant (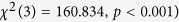. These findings indicated that there was a significant asymmetry between the left and right hemispheres. Table 1 also shows that, in all datasets, the proportion of both the prominent and present PCSs in the left hemisphere was twice that in the right hemisphere. The PCS distribution characteristics in all datasets indicated that the PCS was larger and more common in the left hemisphere than in the right hemisphere.

### Differences with respect to sex

The probability of the PCS presenting in the left and right hemispheres was separately compared in males or females, and the chi-square analyses within the male and female groups were performed separately to study any sex-related differences in the PCS (Table 2). A significant asymmetry of the PCS was identified in both males and females in all datasets (dataset 1 males: 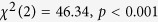, dataset 1 females: 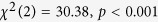, dataset 2 males: 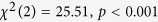, dataset 2 females: 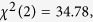

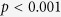
, dataset 3 males: 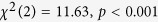, dataset 3 females: 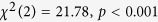, dataset 4 males: 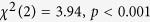, dataset 4 females: 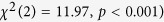. The probability of the prominent and present PCS in the left hemisphere was greater than in the right hemisphere in both males and females for all four datasets (Table 2). These findings indicated leftward lateralization of the PCS in both males and females.

### Effects of handedness

Dataset 4 contained 41 subjects who were left-handed, so this dataset was used to study the effects of handedness on the three types of PCSs - prominent, present, and absent. The data was analyzed without taking into consideration any sex effects. The statistical result (dataset 4: 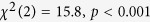) identified an significant asymmetry of the PCS between the left and right hemisphere. Specifically, the proportion of prominent PCSs was 5% in the left hemisphere, compared with 0% in the right hemisphere. The statistical results about the appearance of the PCS and the percentages for the prominent, presence, and absence of the PCS occurrences in the left and right hemispheres in each datasets that are shown in Table 1 indicate that left-handers have the same leftward lateralization of the PCS as right-handers.

### Interactive effect between handedness and sex in the morphology of the PCS

The distribution of the prominent, present, and absent PCSs in the left hemisphere was studied between males and females (Table 3). In dataset 1 the frequency of a prominent PCS in the left hemisphere in males and females was 24% and 9%, respectively. In dataset 2, the frequency of a prominent PCS in the left hemisphere of males and females was 10% and 8%, respectively. In dataset 3 the frequency of a prominent PCS in the left hemisphere of males and females was 12% and 9%, respectively. These finding showed that right-handed males were more likely to have a prominent PCS in the left hemisphere. In dataset 4, the frequency of a prominent PCS in the left hemisphere in left-handed males and females was 10% and 16%, respectively (Table 3). This result showed that, unlike the right-handed subjects, left-handed females were more likely to have a prominent PCS in the left hemisphere.

### Effects of race

Datasets 1 and 2 were Chinese subjects, all the subjects in dataset 3 were German, and all the subjects in dataset 4 were Belgian. As Table 1 shows, the chi-square statistical finding for each dataset identified a significant asymmetry of the PCS in the left and right hemispheres. All the datasets also showed a leftward lateralization of the PCS (the PCS was more often prominent and present in the left hemisphere) in both males and females (Table 2). These results show that the presence of leftward asymmetry of the PCS was not affected by racial differences.

## Discussion

In our current study, we examined structural magnetic resonance images to study whether the morphology of the PCS was affected by sex, handedness, or race. The initial studies^5^ investigated the morphical characteristics of the PCS based on autopsies, and the sample was limited. Our current study used a large sample of four different datasets to study whether the morphical characteristics of the PCS are affected by sex, handness, or race. The main findings of our study are:

1. The PCS was more frequently prominent or present in the left hemisphere and more frequently absent in the right hemisphere. We called this phenomenon the left lateralization of the PCS.
2. There were no significant sex-related differences of the PCS in the left lateralization, but the distribution of the prominent PCSs in the left hemisphere differed between males and females.
3. The leftward asymmetry of the PCS was not affected by race.
4. There were no significant effects of handedness on the left lateralization of the PCS, but handedness affected the distribution of the prominent PCSs in the left hemisphere in males and females.

### Asymmetry of the PCS

In our study, by comparing the PCS properties in the left and right hemispheres in all the datasets, we found an asymmetry of the PCS between the left hemisphere and the right hemisphere and identified a left lateralization of the prominent and present PCSs. This finding was consistent with previous findings in post-mortem^5^,^12^ and magnetic resonance imaging (MRI) based studies^10^,^11^. Our finding also agrees with previous results from clinically-related studies of schizophrenia. Previous studies of the ACC morphology of schizophrenic patients found a reduced degree of leftward asymmetry of the PCS^20^,^21^. Also, the presence or absence of a PCS seemed to influence the activation of the left anterior cingulate cortex in a group analysis of schizophrenia patients^16^. In addition, a study by Le Provost and colleagues reported that alterations in the PCS may be associated with vulnerability to schizophrenia^21^.

Regional differences in connectivity profiles may result from variations in cortical folding, which may be induced by developmental changes in axonal tension between connected brain areas, indicating that strong connections in the cortex play an important role in the formation of gyri^22^,^23^. Postmortem research showed that, when the PCS was present, it always contained a large part of Brodmann’s area 32 (BA32), when the PCS was absent, BA32 was always buried on the dorsal bank of the CS, so both cytoarchitecture and connectivity may result in differences in the morphological properties of the PCS^5^. Thus, the morphological differences in the PCS may be related to tissue distribution and the connection modes of the left and right hemispheres.

Many previous studies have revealed that individual differences in perception and mental functioning are related to the PCS morphological lateralization. Neuropsychological studies have found that differences in the CS and PCS morphology are related to cognitive performance^15^,^24^. For example, schizophrenic patients and young men at high-risk of developing a psychotic illness showed a substantial reduction in sulcal asymmetry in the ACC^20^,^21^. Another previous study^15^ also demonstrated that the PCS asymmetry was related to both spatial working memory (SWM) and verbal fluency (VF). Thus, structural lateralization may underlie the observed functional asymmetry in this area.

### Effect of sex

The anatomical asymmetry of many brain areas has been found to be greater in males than in females in healthy subjects^25^,^26^. Our current study found that present PCSs were left asymmetric in both males and females and the left asymmetry of the PCS’s morphological properties showed a significant sex effect. This finding in the right-handed subjects is consistent with previous findings that reported that males have a more fissured AC (indicating a prominent PCS) in the left hemisphere^10^. Also, the results of a previous MRI study, which measured the cingulate gray matter volume and found a leftward asymmetry in males but not in females^27^, were similar to ours. A study by Paus and colleagues found a higher incidence of the two extreme categories of the PCS in females but more average categories of the PCS in males^11^, indicating a sex effect on the morphological properties of the PCS. Another study showed that the PCS asymmetry changed at different ages in males and females^18^, suggesting that the PCS exemplifies a sex-dependent trajectory of cerebral asymmetry in which the male brain matures more slowly and reaches a plateau later than the female brain^28^. Combined with previous studies, our findings may support the Leonard and colleagues’ study result that males have a larger cerebral volume than females^29^. However, in Leonard and colleagues’ another study^30^, they found that men showed no obvious asymmetry, whereas women showed highly significant asymmetry even 26 left-handed subjects excluded. And Leonard *et al*.^30^ concluded that sex difference may be due to the men had twice as many overlapping and prominent PCS in the right hemisphere as the women. Their findings were inconsistent with all the previous studies of right handers^6^,^10^,^11^,^17^ and were also different with our findings. The main difference between our findings on sex factor and Leonard and colleagues’ study^30^ may result from different definition of the anterior border of the PCS or different depth of the observed lateral slices in the respective hemispheres. The difference may also be due to the interaction between sex and handness.

### Effects of handedness

Our study found that, in both the right- and left-hander datasets, the males and females both had a greater frequency of PCSs and that the PCSs tended to be more pronounced in the left than in the right anterior cingulate region.This finding indicated that handedness does not affect the presence, absence, or prominence of the PCS in the trend toward leftward asymmetry. We also found that the right-handed males had a higher number of prominent PCSs in the left hemisphere than the right-handed females, but the opposite was found in left-handed females. Our finding, which identified the main effects of handedness and sex on the percentage of prominent PCSs in the left hemisphere, was similar to a previous study’s results^17^. The PCS is also known to affect cortical folding patterns. Huster and colleagues revealed that in left- or right-handed males and females the ACC displayed a bias toward leftward asymmetric folding patterns, as opposed to symmetric or rightward folding patterns, and that discrepancies from this general rule were diminished in male left- and female right-handers^17^. Since previous studies have reported that PCS variability affects cortical morphometry, including cortical thickness, surface area, volume, and sulcal depth in healthy humans^8^,^9^, our finding about the effects of handedness and sex on the percentage of prominent PCSs in the left hemisphere may to some extent explain Huster and colleagues’ results.

### Effects of race

Cultural psychologists have shown ample evidence for differences in cognition and behavior between East Asian and Western cultures^31^,^32^. Cultural neuroscience studies that compared fMRI results between East Asians and Westerners or that primed participants with East Asian or Western cultural values have shown cultural differences in neural correlates of cognition and behavior in that culturally influenced activations were found in the higher-order cortices (frontal, parietal, temporal) that are associated with cognitive control, attention, and working memory^33^,^34^,^35^. A recent study^36^ suggested that cultural differences in social and non-social processes are mediated by distinct neural networks. Neuropsychological studies have found that differences in the CS and PCS morphologies are related to cognitive performance^15^,^24^. A recent study also indicated that different neural mechanisms may underlie racial differences in responses to armed versus unarmed targets^37^. In our study, we found a left lateralization in both Asian and European populations. This finding indicated that there are no significant racial differences in the presence, absence, or prominence of the PCS.

## Materials and Methods

### Subjects

Four independent datasets of the MRIs of healthy subjects were used in our current study. Datasets 1 and 2 were in-house MRI datasets, and datasets 3 and 4 were respectively acquired in Germany and Belgium. Dataset 1 included 332 (169 males and 163 females; mean age, 19.1 years; range, 17–27) healthy right-handed subjects who were recruited from the University of Electronic Science and Technology of China. Dataset 2 included 326 (157 males and 169 females; mean age, 23.1 years; range, 19–25) healthy right-handed subjects who were recruited from Tianjin Medical University. The MRI data in dataset 3 included 128 (60 males and 68 females; mean age, 39.8 years; range, 20–68) healthy right-handed subjects who were scanned in Germany. Dataset 4 was obtained in Belgium and included 41 healthy left-handed subjects (10 males and 31 females; mean age, 20.5 years; range, 18–30). None of the participants had ever suffered from any psychiatric or neurological disease, and none had any contraindications for MRI scanning. All the participants signed a local informed consent before MRI scanning, and all the experimental procedures and MRI scanning were approved by the local ethics committees of University of Electronic Science and Technology of China, Tianjin Medical University, Research Centre Jülich, and Ghent University Hospital, respectively. And all the experiments and the methods were performed in accordance with the approved guidelines and regulations.

### MRI data acquisition

Both datasets 1 and 2 were acquired using a 3.0T GE Signa MRI scanner. For dataset 1, sagittal 3D T1-weighted images were acquired with the following parameters: TR/TE = 8.16/3.18 ms; inversion time = 800 ms; FA = 7°; FOV = 256 mm × 256 mm; matrix = 256 × 256; slice thickness = 1 mm, no gap; 188 sagittal slices. For dataset 2, the scan parameters for the sagittal 3D T1-weighted images were as follows: TR/TE = 8.1/3.1 ms; inversion time = 450 ms; FA = 13°; FOV = 256 mm × 256 mm; matrix = 256 × 256; slice thickness = 1 mm, no gap; 176 sagittal slices. For dataset 3, a structural scan was acquired using 3 T Simens MRI scanner for each participant with the following parameters: 176 axial slices, TR = 2.25 s, TE = 3.03 ms, FOV = 256 × 256 mm2, flip angle = 9°, final voxel resolution: 1 mm × 1 mm × 1 mm. The dataset 4 structural T1 images were collected also using 3 T Simens MRI scanner with the following parameters: TR = 1,550 ms, TE = 2.39 ms, image matrix = 256 × 256, field of view (FOV) = 220 mm, flip angle = 90°, voxel size = 0. 9 mm × 0. 9 mm × 0.9 mm.

### Classification of PCS

#### Classification criteria

**Figure 1:**
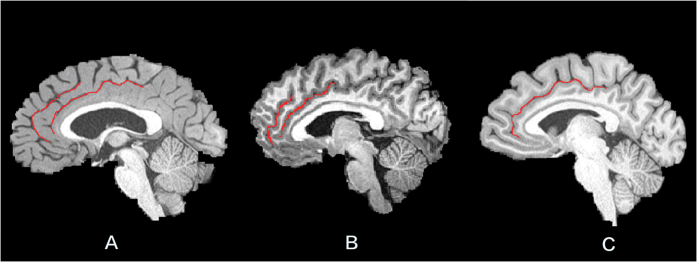
The variations in the paracingulate sulcus (PCS) and the cingulate sulcus (CS) morphology. The red lines represent the the cingulate sulcus (CS) and the paracingulate sulcus (PCS). The bottom red line represents CS, and the top red line represents PCS. (**A**) A prominent PCS was shown in the left hemisphere, (**B**) A present PCS was shown in the left hemisphere, (**C**) An absent PCS was shown in the right hemisphere.

The appearance of the PCS was analyzed based on the structural MRI images according to the criteria proposed by Yucel and colleagues^10^ and was classified into three categories: prominent, present, and absent. This was done in two steps. First, we determined whether there was a PCS. To identify the PCS, we observed whether there was a sulcus parallel to the CS. Second, if there was a sulcus parallel to the CS, we classified the sulcus into three categories: prominent, present, and absent. The criteria for this classification were: If the length of sulcus was longer than 40 mm, we considered that this sulcus was ‘prominent.’ If the length of the sulcus was between 20 mm and 40 mm, we considered this sulcus to be ‘present.’ All other conditions were classified as ‘absent.’ The PCS is parallel to the CS; thus, before assessing the PCS, we had to assess the CS. Its morphology was assessed based on three important properties^7^: (a) The CS passes from the front of the corpus callosum to its knee. (b) The CS is located deep within the brain rather than at the surface. And (c) the CS stretches upward and borders with the marginal sulcus. The three types of PCS were showed in Fig. 1.

#### Definition of the paracingulate sulcus

The PCS was defined by observing whether the sulcus was running dorsally and parallel to the CS, as described in a previous study^10^. In our current study, we used MRIcron software (http://www.softpedia.com/get/Science-CAD/MRIcron.shtml) to analyzed individual anatomical images. After identifying the PCS, on the basis of the length of the sulcus, the sulcus was classified into three categories, prominent, present, and absent according to the classification criteria described by Huster and colleagues^17^. First, we delineated the border of the relevant region of interest (ROI) using the criteria adopted by Yucel and colleagues^10^. The anterior border of a sulcus was defined as the turning point, which was specified by lines running perpendicular to the anterior/posterior commissural plane (AC–PC plane) of its ventral to dorsal course^10^. The posterior boundary of the ROI was defined by another line perpendicular to the AC–PC plane but crossing the anterior commissure^10^. Second, a protocol for classifying the ACC that would help to ensure the reliability of these ratings was generated. Starting at a mid-sagittal slice, the PCS, which was defined as a sulcus running dorsal and parallel to the CS, had to be certified on at least three more lateral slices in the respective hemisphere. This was done to avoid confounding the hemispheres in the mid-sagittal view and to distinguish the PCS from other, often more superficial sulci, such as the intralimbic sulcus. If present, the extension of the PCS was registered voxelwise yielding a measure in millimeter resolution. Interruptions or gaps in the PCS course did not contribute to the length measurements^10^.

### Statistical analyses

After obtaining the quantitative description of the morphological characteristics (prominent, present, or absent) of the PCS in the left and right hemispheres, a chi-square test (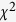) was used on each dataset to test whether the morphological characteristics of the left and right PCS were asymmetric. The chi-square tests (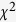) were performed separately in the males and the females to investigate whether the asymmetry of the leftward and rightward PCSs was impacted by sex. McNemar’s test for symmetry was used on the overall sample to test whether the number of cases of PCS asymmetry in one direction was counterbalanced by an equal number of cases with an asymmetry in the other direction. All the statistical analyses were performed in SPSS software.

## Conclusion

In this study, we demonstrated that the PCS was more frequently prominent in the left hemisphere and more often absent in the right hemisphere. Sex-related differences, racial differences, and handedness differences did not affect this phenomenon. Handedness primarily affected the frequency of prominent PCSs in the left hemispheres of both males and females but in opposite directions. These findings may facilitate future studies correlating the morphology of the PCS with cognitive behavior.

## Additional Information

**How to cite this article**: Wei, X. *et al*. Paracingulate Sulcus Asymmetry in the Human Brain: Effects of Sex, Handedness, and Race. *Sci. Rep.* **7**, 42033; doi: 10.1038/srep42033 (2017).

**Publisher's note:** Springer Nature remains neutral with regard to jurisdictional claims in published maps and institutional affiliations.

## Figures and Tables

**Table 1:** Hemispheric differences in the morphological characteristics of the PCS.

Left hemisphere	Right hemisphere				Analysis
	Prominent	Present	Absent	Total	
Prominent	9 (2)	11 (3)	34 (10)	54 (16)	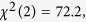 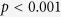
Present	6 (2)	28 (8)	106 (32)	140 (42)	
Absent	5 (2)	25 (8)	108 (33)	138 (42)	
Total	20 (6)	64 (19)	248 (75)	332 (100)	
Dataset 2 (right handed)					
Prominent	0 (0)	7 (2)	22 (7)	29 (9)	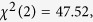 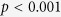
Present	1 (0.5)	7 (2)	74 (22.5)	82 (25)	
Absent	1 (0.5)	26 (8)	188 (57.5)	215 (66)	
Total	2 (1)	40 (12)	284 (87)	326 (100)	
Dataset 3 (right handed)					
Prominent	0 (0)	2 (2)	11 (8)	13 (10)	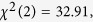 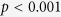
Present	1 (1)	4 (3)	32 (25)	37 (29)	
Absent	1 (1)	3 (2)	74 (58)	78 (61)	
Total	2 (2)	9 (7)	117 (91)	128 (100)	
Dataset 4 (left handed)					
Prominent	0 (0)	1 (3)	5 (12)	6 (15)	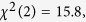 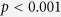
Present	0 (0)	0 (0)	11 (27)	11 (27)	
Absent	0 (0)	1 (2)	23 (56)	24 (58)	
Total	0 (0)	2 (5)	39 (95)	41 (100)	
Overall					
Prominent	9 (1)	21 (2)	72 (9)	102 (12)	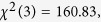 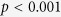
Present	8 (1)	39 (5)	223 (27)	270 (33)	
Absent	7 (1)	55 (7)	393 (47)	455 (55)	
Total	24 (3)	115 (14)	688 (83)	827 (100)	

The number of the subjects who had a prominent PCS, a present PCS, or the absence of a PCS in the left and right hemispheres in each dataset.

Percentages (%) are given in parentheses. Prominent: prominent PCS, present: present PCS, absent: absent PCS. Total: the number of participants in each dataset, ‘left’ and ‘right’ mean left and right hemispheres respectively. Overall: the sum of the subjects in all four datasest. Analysis: the results of statistical tests.

**Table 2:** Hemispheric differences in the PCS morphology in males and females.

	Dataset 1 (right handed)			Dataset 2 (right handed)			Dataset 3 (right handed)			Dataset 4 (left handed)					
	Prominent	Present	Absent	Prominent	Present	Absent	Prominent	Present	Absent	Prominent	Present	Absent			
Male															
Total	169(100)			157(100)			60(100)			10(100)					
Left	40(24)	70(41)	59(35)	15(10)	40(25)	102(65)	7(12)	13(22)	40(66)	1(10)	4(40)	5(50)			
Right	14(8)	34(20)	121(72)	0(0)	21(13)	135(87)	1(1)	4(7)	55(92)	0(0)	1(10)	9(90)			
Analysis	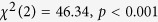			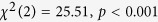			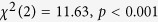			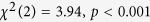					
Female															
Total	163(100)			169(100)			68(100)			31(100)					
Left	14(9)	70(43)	79(48)	14(8)	42(25)	113(67)	6(9)	24(35)	38(56)	5(16)	7(23)	19(61)			
Right	6(4)	30(18)	127(78)	2(1)	19(11)	148(88)	1(2)	5(7)	62(91)	0(0)	1(3)	30(97)			
Analysis	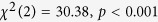			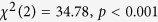			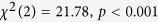			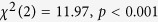					

The number of subjects with different PCS morphologies in the left or right hemisphere in males and females in each dataset. Percentages (%) are given in parentheses. Data, in the upper panel of the table, are the numbers of subjects with differences in the PCS morphology between the left and right hemispheres in males. Data in the lower panel of the table are the numbers of subjects with differences in the PCS morphology between the left and right hemispheres in females. ‘Total’ means the sum of the male and female participants in each dataset; ‘left’ and ‘right’ mean the left and right hemispheres, respectively. Analysis: the results of statistical tests.

**Table 3:** Gender and handedness effect in the distribution of prominent PCSs in the left hemicerebrum.

	Prominent	Present	Absent	Total
Dataset 1 (right handed)				
Male	40(24)	70(41)	59(35)	169(100)
female	14(9)	70(43)	79(48)	163(100)
Dataset 2 (right handed)				
Male	15(10)	40(25)	102(65)	157(100)
Female	14(8)	42(25)	113(67)	169(100)
Dataset 3 (right handed)				
Male	7(12)	13(22)	40(66)	60(100)
Female	6(9)	24(35)	38(56)	68(100)
Dataset 4 (left handed)				
Male	1(10)	4(40)	5(50)	10(100)
Female	5(16)	7(23)	19(61)	41(100)

The number of subjects with differences in the PCS morphology in the left hemisphere in the males and females in each of the datasets. Proportions (%) are given in parentheses.
